# Supplementary material for: Patterns of Intron Gain and Loss in Fungi
Source: PLoS Biol. 2004 Nov 30;2(12):e422. doi: 10.1371/journal.pbio.0020422 (PMC532390; doi:10.1371/journal.pbio.0020422)
Supplement: Table S1 — Also available at http://genes.mit.edu/NielsenEtAl/. (4.3 MB ZIP). [file pbio.0020422.st001.zip › NielsenEtAl/html/958.html]

AN0850.1.NCU00167.1.MG11100.1.FG06643.1


```
 CLUSTAL W (1.82) Multiple Sequence Alignments - Introns Inserted


Sequence 1: NCU00167.1	636 aa
Sequence 2: FG06643.1	618 aa
Sequence 3: MG11100.1	655 aa
Sequence 4: AN0850.1	621 aa
Alignment Length: 715 aa
Number Identitical Residues: 168 aa
Alignment Score (without introns) 9377


MG11100.1 	-MTSRPAPTPISAPKP-----ALLSDLPPVSALFLIEFDVKAG~YTITWKKAAPGI~SLD
NCU00167.1	MTPYRGRQPSLSLRLPQPSASAVLPDLPPISALFLIDFDVKAG~YTIVWKRSVPGL~ELD
FG06643.1 	MATSRRGQPSLSVRMP----SNQPTDRPPIDALFLIHFDVKAG~YTILWKQNAPGI~DLD
AN0850.1  	MGRPSVQRKSLGLDVPR---TTAAEALPGIAALFVIHFDVKAG2YTVTWKRTIPGV1EVE
          	         .:.   *    :      * : ***:*.****** **: **:  **: .::

MG11100.1 	GAVEYKSLPSGLHTVSQDLI~YFIHEGEYAGLSAFINAKTEEEATRNARMIAVGVLVPLS
NCU00167.1	GVVEYKSLPSGLHTVTDDLV~YFVHEGGHAGLSAFVNARTDEEENRHARMIAVGVLVPLV
FG06643.1 	GLVEYKSLPSGLHTVSEDLI~YFVHDSVHAGLSAFVNIPCDEEEARNARMFAVGVLVPLS
AN0850.1  	GAVEYKSLPSGLHNVSEDLV2YFVHD-QYAGISAFVNQPADESE-RNAKMFSIGVLVPLS
          	* ***********.*::**: **:*:  :**:***:*   :*.  *:*:*:::****** 

MG11100.1 	YGRLGRSWRHAEGLREMAM~KLAVENKDLSILETYWEKNKAHDSMSPSAEPPKESPPSSP
NCU00167.1	YGRLGRSWRHADRLKDIAS2KLVVDRQQTHLLEDYWAQNGIQDGAHPPPLP--EAALDSP
FG06643.1 	FGRLGRAWRHAEGLKEMAA2KLAEDRKNTSILDEYWVANREKAGNGNRRLSG-ETPVSSP
AN0850.1  	SGRLGKSWRHASKLKELAQ2RYSESAFSTNSLSEYWETHELSAN-ETSAAP--DSPLDSP
          	 ****::****. *:::*  :   .  .   *. **  :    .      .  ::. .**

MG11100.1 	SVTF---QPGATQSQFRKRTGSDAQALVPPGHQLSPFHPAWSLSDLLDTFGPLIFPIHRA
NCU00167.1	ALSYKGGRPGPGKGHSRNRSASDGTVLMSSPHRLSPYHPAWSLTSLLDTFGPLIFPIHRA
FG06643.1 	SLTS---QPFHRKAHNRNRSASDGAALISPGNKLSPYHPAWSLTSLLDKFGPLIFPIHRA
AN0850.1  	LSLRMRAHSDRPDTLQRNRAISDAMVLETSRPALTPFHPASSLPDFLDAFGPLLFPLYRA
          	      .:.   .   *:*: **. .* ..   *:*:*** **..:** ****:**::**

MG11100.1 	ALLRKRILISTYAPVHQACDFV1YDISILSNIPLSLFDLLDP-TAPAQRLRPLFNIGVND
NCU00167.1	ALLRKRILISTHAPVHEACNFV1YNLSVLSNIPLSVSDLVDP-AASTQRLRPLFNIGVHD
FG06643.1 	ALLRKRILISCHAPVHEVCDFV1YNMSVLSNIPLSVIDILPG-STSTQRLRPLFSIGVHD
AN0850.1  	ALLRRRVLFMAEAPVQVPCNYV1YDLSLLASLPNSLLPLLPSDRLLSLRPRALFNVGVYD
          	****:*:*:   ***:  *::* *::*:*:.:* *:  ::  .   : * *.**.:** *

MG11100.1 	IAFLSEDAAAAVSKKRCASSASASHSEQSSLPEQAAGSGWVACTTDSILAMKPDLWDMLI
NCU00167.1	IGYLLEHHAAMK-RHLRGDNLETDNDLASSITAEEAATGWIACTTDTILTIKDGLWDMLI
FG06643.1 	IPFLMDDFEASK-KKQSDEDVIDD----------ESGSGWIACTTDSILAMKDTLWDMLI
AN0850.1  	IPYLSSFIRGSA--------------------DSEHDSTWIACSTDSVLSMKPELFDVLV
          	* :* .   .                       .   : *:**:**::*::*  *:*:*:

MG11100.1 	TMPAPHTAGAKERVWPTIEL---------PGHVPVRATQRDLRRFRSLRAGIIRLAR---
NCU00167.1	TLPPPHSGNAKERMWPTVEC---------PKGVPIKATQRDLRRFKWLKMSLARIDPKSM
FG06643.1 	TLPPDYSSNATERVWPLVEC---------PREQPVKATQRDLRRFTSLKNGLARLEAITQ
AN0850.1  	TLPPSHTKNAASRAYPTISVSSPLVDKKPPQRTELKATQRDVRRYIALRKGLRRVFK---
          	*:*. :: .* .* :* :. ::.  ....*    ::*****:**:  *: .: *:     

MG11100.1 	--PMSARSERPGTSSGWS-------------------------AATAVAKSDDDSLLADA
NCU00167.1	TTPQILRSPLSDRTSPRSVSTPNVPNRTPTISLTRPTETSRPVTATSQTPPLTRANSDDD
FG06643.1 	APSMVPESPEEERFARR---------------------------VSTHSNHVVRDPRDDA
AN0850.1  	--DECIRS-------------------------------------NSDEDDSSDAASTFS
          	      .*                                     .:             

MG11100.1 	VDKVVEPTTWAALAYSGFMWWASAGEQR-----RSEEAEEVAQDSSLLADLASAPSPRRR
NCU00167.1	AERVVEPTTWAALAYSGFIWWASAGEQG-----HSDEVDELAHDSALLADLTGN------
FG06643.1 	VEKVVEPLTWAAFAYNGYMWWASAGEQL-----RSEQQEEAAHDASLLADFGHS------
AN0850.1  	SSPIVEPLSWTRLAYTSFIWWASAGEKRDGFSEEEEEEHEVEQDARLLASVDNIPSP---
          	 . :*** :*: :**..::*******: .. :...:: .*  :*: ***..   .:.   

MG11100.1 	GSSVSGGVSGHLAKNIAVMADSVMSLPAAGQMNIGDSDVAAIEAPSQQSELG-DAVDHTE
NCU00167.1	------------TPKMAGRTHRRTSFGTASLIGNNLEEGGITESDSSPTAQQDGAREISD
FG06643.1 	------------SMTMP-RPGSRSEQ--------------LSDSLSSLNATR---RANDD
AN0850.1  	------------PSGSIRQPNMQQEQTPTQPPEIALIAYFRQLTTQIFVTLSDVIARQDD
          	            .      .    .  .:              : .      .      :

MG11100.1 	EERARVELAIIAYFRRLTTQMLTVLGDLVDSIED-----GDDEEMDETETTEDSP~LNQS
NCU00167.1	EEQARIELAIIAYFHRLTTSVLSVLADLVDSAD-------DDDLLDLDIDPNAGV1K---
FG06643.1 	SGEARFELAIIAYFHRLTTQILSVLADLADSQDEPYTDQDDDDGLSQISDDRTPL~RPSD
AN0850.1  	HSGIDPEAASTISNEDQVENTETFTDDIPDDDPS----AGDDTSPLLEPNRDPQV~KAQA
          	      * *     .  . .  :.  *: *.  .     .**                . 

MG11100.1 	DDGDACLVGSLHVDSDALARMGLDVWSDADVAFVKEATACYFARRARVESKAVEVCGLRV
NCU00167.1	SDPES-----------------------------KVVIVEVKARTTKPSMK--AVCGGG-
FG06643.1 	SDPEPDDADAIRVDSHAVESMGLDVWSDADALFIRELSIKYFSRSARIEGKGVEVCGVRV
AN0850.1  	RDTDAIIIN-----AEDMAEMGLDTWSAADRIFVEELVLTWWGRKAYVDSARIRCCGISI
          	 * :.   .     :      . .  : :.    .       .* :  .      **   

MG11100.1 	C
NCU00167.1	-
FG06643.1 	C
AN0850.1  	I
          	
```
